# Recent Progress in Optical Biosensors Based on Smartphone Platforms

**DOI:** 10.3390/s17112449

**Published:** 2017-10-25

**Authors:** Zhaoxin Geng, Xiong Zhang, Zhiyuan Fan, Xiaoqing Lv, Yue Su, Hongda Chen

**Affiliations:** 1 School of Information Engineering, Minzu University of China, Beijing 100081, China; s151189@muc.edu.cn; 2 State Key Laboratory for Integrated Optoelectronics, Institute of Semiconductors, Chinese Academy of Sciences, Beijing 100083, China; Fanzhiyuan@semi.ac.cn (Z.F.); lvxiaoqing284@semi.ac.cn (X.L.); suyue@semi.ac.cn (Y.S.); hdchen@semi.ac.cn (H.C.)

**Keywords:** biosensor, optical, microscope, spectrometry, smartphones

## Abstract

With a rapid improvement of smartphone hardware and software, especially complementary metal oxide semiconductor (CMOS) cameras, many optical biosensors based on smartphone platforms have been presented, which have pushed the development of the point-of-care testing (POCT). Imaging-based and spectrometry-based detection techniques have been widely explored via different approaches. Combined with the smartphone, imaging-based and spectrometry-based methods are currently used to investigate a wide range of molecular properties in chemical and biological science for biosensing and diagnostics. Imaging techniques based on smartphone-based microscopes are utilized to capture microscale analysts, while spectrometry-based techniques are used to probe reactions or changes of molecules. Here, we critically review the most recent progress in imaging-based and spectrometry-based smartphone-integrated platforms that have been developed for chemical experiments and biological diagnosis. We focus on the analytical performance and the complexity for implementation of the platforms.

## 1. Introduction

With significant improvement of hardware and software, smartphones, which are convenient and as powerful as computers, have become necessary for everyone. Applications (APPs) of smartphones were extended and updated quickly. Especially, smartphones have been widely used in biosensing assays, acting as detectors, data processors, and even signal inducers with an additional custom designed cradle or attachment which was used to mount other small optical or other components [1,2,3,4]. Therefore, they were potentially more accessible and cheaper than portable analytical laboratory devices in routine clinical tests, where they has been increasing interest in using smartphones to detect analytes of clinical interest. Various biologically derived materials, such as enzymes, cells, nucleic acids, antigen–antibodies, and microorganisms, which could be tested on smartphones. Currently, many researchers have focused on how to combine smartphones and micro-biosensors, micro-bioelectronic mini-devices, microfluidic chips together to achieve real-time, point-of-care, and easy-to-use detections for analytes, especially for clinic and environmental samples [1,2,3,4,5,6,7,8,9,10].

Meanwhile, portable clinical diagnostics-based smartphone platforms have grown into a blooming market with significant growth expected, especially in remote and rural areas. The demand for accessible point-of-care testing (POCT) technologies has been rapidly increasing due to the shortage of portable testing resources. This has created unexpected opportunities for expanding the diagnostic and the prognostic approach to a large number of pathologies and for evaluating pharmaceutical therapies. With the aid of 3D-print technology, smartphone-based systems with 3D-printed cradles which were used to fix attachments have been explored in diagnostic field [8,9,10], food safety [11,12], environmental monitoring [13] and physical condition [3,14]. Though there are many different biosensor platforms based on smartphones, high-quality camera lenses on smartphones are a critical part and applied extensively for different smartphone biosensor platform. By using the photocameras of smartphones as a ‘smart detector’, complementary metal oxide semiconductor (CMOS) image sensors acting as a ‘smart recorder’, specific optical APPs acting as ‘smart readout’, almost all the optical-based testing methods such as imaging, absorbance, reflectance, fluorescence, surface plasmon resonance (SPR), localized surface plasmon resonance (LSPR), bio-chemiluminescence and electrochemiluminescence could been integrated together. Especially, in color-intensity-based assays, an attachment is often needed, which helps the smartphone convert the presence and concentration of a target analyte into colorimetric changes that could be measured by the smartphone camera [5,6,7]. The signal transduction of color-intensity-based smartphone biosensor platform, whether based on immune complex formation or a specific chemical reaction, generally involve an assessment of color changes as the final result in an assay. Therefore, CMOS image sensors of smartphone are used to collect the optical signals, then an algorithm, which is applied in Android or IOS (the Apple iPhone operation system) APPs, is used to process the images captured by the CMOS detector. With that, the testing results display on the screen of smartphone. In testing image-analyzing step, an effective algorithm is critical to the speed and the precision of bio-test-platforms based on smartphones.

Though there are several reviews on biosensors based on smartphone platforms [15,16,17,18,19], portable smart devices and flexible biosensors based on optical methods have developed very fast. Therefore, here, we critically and mainly review the most recent progresses that have used smartphones based on optical methods as analytical devices and biosensors. We would classify the recent smartphone-based biosensing researches by the imaging and spectrometry functions of smartphones, and discuss the benefits and drawbacks of the assays performed by different groups in recent work.

## 2. Biosensor Based on Imaging

In the hospital, many diseases are diagnosed under the microscope, such as bacterial infections, blood-borne diseases and tumor cell detection. Microscopes were used to detect objects which were too small to be seen by the naked eye. The images of small objects were magnified by a series of lenses in the microscope. However, the cost and bulkiness had limited its applications in resource-poor areas. Portable and low-cost microscopes were in high demand. In 2007, Frean et al. demonstrated that cameras on mobile phones were able to capture photos from the eyepiece lens of a microscope [15]. Inspired by Frean’s work, many research groups began to use smartphones to form imaging systems for bio-detection [1,16,17,18]. With the help of the simple optics and the CMOS of the smartphone, a variety of smartphone-based imaging systems were developed to meet the demands of various detection problems.

### 2.1. Microscopes

In general, the smartphone-based microscope has an extra attachment which consists of a series lenses and other optical components. These attachments serve as microscopes which produce the images of analytes. Smartphones serve as cameras that capture the images and store them in digital form. The basic structure of the smartphone-based microscope is easy to build up based on the principle of microscopes. However, various efforts have been made to improve the quality of the smartphone-based microscopes.

In 2009, Breslauer et al. proposed a mobile phone-based microscope using a long lens system [20]. The lens system included almost all elements of a typical microscope, such as a light-emitting diode (LED) light source, collector and condenser lens, a sample stage, an objective, an eyepiece and several filters which were used for fluorescent detection (Figure 1.1). This was a typical example of a smartphone-based microscope.

The smartphone used in Breslauer’s work was a Nokia N73, which was equipped with a 3.2-megapixel (2048 × 1536 pixel) camera from the Carl Zeiss Jena, a company famous for producing camera lenses. With the help of attachments and the good CMOS camera, the resolution of this imaging system could reach 1.2 μm, which was thought as a slightly conservative estimate. With the developing computing function of smartphones, some imaging techniques as well as algorithms were applied to the smartphone based microscope. In 2013, Navruz et al. proposed a smartphone-based computational microscopy [21]. In this system, a rotatable fiber-optic taper and a two-lens imaging system were coupled (Figure 1.2). The samples were in contact with the top of the fiber-optic taper and illuminated by an extra LED. When the fiber-optic taper was rotating manually, images of different degrees were stored in memory of the smartphone. These images would be processed by a custom-develop APP running on the Android system. Finally, the image of the sample could be presented on the screen of the smartphone with a spatial resolution of 1.5–2.5 μm. The authors believed that the spatial resolution of this system could be improved using a denser fiber-optic array, potentially reaching sub-micron level. Compared with Breslauer’s work, this system was more compact and convenient. Meanwhile, the APPs made this system more simple to use.

Recently, microfluidic chips have become a research hot-spot due to the fact they are inexpensive and require ultra-small sample volumes. These advantages of microfluidic chips were taken advantage of in a smartphone-based microscope to count markers. In 2017, Kanakasabapathy et al. reported a rapid and simple smartphone-based microfluidic chip for CD4 testing [22]. The microfluidic chip was fabricated using polymethylmethacrylate (PMMA) with a 40 mm × 5 mm microchannel (Figure 1.3). A small volume of blood, about 30 μL, was injected into the microchannel which had been functionalized with anti-CD4 antibodies beforehand. The microchannel was washed with phosphate buffered saline (PBS) solution after CD4 were captured by the antibodies. Finally, the image of the microchannel along with the CD4 cells was captured by the smartphone with the help of some extra lenses. A custom-developed software was applied to analyze the image and count the number of cells. The authors stated that the potentially resolution of the system was 33 cells per μL because 1 cell per field of view could be detected. In practice, the system had a sensitivity of 100% and specificity of >90% at 200 and 500 cells per μL thresholds which are competitive with commercially available devices. Besides, the cost of this system was much lower than that of the commercial devices. The cost of the optical smartphone attachment was less than 5 dollars and the cost of the microfluidic chip was less than 4.75 dollars, including materials and production cost, while the commercial devices cost thousands of dollars. Meanwhile, detecting and counting fluorescence markers is a typical method in biosensing. The smartphone-based fluorescence microscope was designed by Zhu et al. [23,24] for instance, in [24], a microfluidic device, a color filter, a lens and a LED were assembled compactly (Figure 1.4). A video was recorded by the smartphone when fluorescence markers flowed through the microfluidic channel. After digital processing of the video, the number of the markers could be counted accurately.

Phase microscopes are popular devices which could obverse the cell with more stereo feeling. In 2007, Meng et al. presented a smartphone-based quantitative phase microscope [1]. The authors used a quantitative phase imaging technique to enhance the imaging contrast. Compared to other quantitative phase contrast techniques, quantitative phase imaging methods have more advantages. First, they provide quantitative cellular phases which is crucial to post-image processing. Second, the authors used the transport of intensity equation (TIE) which did not depend on the coherent illumination and lower the requirement for the light environment. By taking advantage of the smartphone built-in LED, a custom-designed shell, a smartphone and some optics, including a micro-objective and eyepiece, the authors made a quantitative phase microscope (Figure 1.5). To demonstrate the performance of device, a commercial human red blood cell smear, a pap smear, monocot root and broad bean epidermis sections were used. The results showed that this device was capable of high-accuracy quantitative intensity and phase imaging.

### 2.2. Lens-Free Microscope

The lens-based microscopes mentioned above rely on the quality of the lens. Besides, a high-quality optical lens was more expensive than other attachments. Therefore, some researchers have perform studies on lens-free microscopes using various theories. These lens-free microscopes could be much cheaper because they used cheap or even no attachments rather than those expensive optical lenses.

Among these researchers, Ozcan and his group at UCLA have done lots of works on lens-free microscopes [25,26,27]. Tseng et al. presented a lens-free microscopy on a cellphone to reduce the both size and weight of the attachment [27]. An aperture and a LED were used to create hologram of micro-objects based the theory of light interference (Figure 2.1).

Using an algorithm, the reconstructed microscopic images were comparable to those of regular microscopes. However, because of the limitation of computing power of these old devices, analysis was carried out on the computer. In 2014, Lee and Yang proposed a smartphone-based chip-scale microscope [28]. They removed the lens-module in a smartphone and placed the sample on the surface of the image sensor (Figure 2.2). The image sensor could capture the shadow images of samples. Besides, an APP running on a smartphone was developed to analyze the images from the camera owing to the fast development of the computing power of smartphones. With the help of APPs, biosensing could be simpler for people. In 2014, Guan et al. came up with an application of blood typing based on paper sensor and smartphone (Figure 2.3) [29]. A paper sensor used the anti-A, anti-B and anti-D antibodies to identify eight ABO/RhD blood types. The length of positive and negative assays was significant different. The smartphone was used to take a photo of the paper sensor. After analyzing the length of three assays, the smartphone could give out the result of blood type on the screen, which was simple and fast.

### 2.3. Summary

Optical imaging techniques for point-of-care diagnostics based on smartphones is a popular topic [16]. The smartphone-based microscope systems have developed quickly in the past decades. With the help of CMOS cameras integrated in smartphones, these systems could reach a high resolution that are competitive to microscopes in the lab. Depending on various theories, lens-dependent and lens-free microscopes based on smartphones could achieve the same functions, imaging the micro-objects. The comparion of all the biosensor based on imaging on smartphone platform is presented in the Table 1.

However, to improve the resolution of these smartphone-based microscopes, APPs must be developed. For example, the shadow imaging technique mentioned above was very dependent on the algorithm used to reconstructed the photos [28]. Since the attachment is always an extra item which is unwelcome to people, the prospect of the smartphone-based microscope system may lie on the CMOS camera and the algorithm. On the one hand, manufacturers of smartphones should pay more attention to developing smartphone-based healthcare applications. On the other hand, researchers could come up with new theories or algorithms to take full advantage of the CMOS cameras on smartphones.

## 3. Biosensor Based on Spectrometry

In biosensing, there are many analytes that could not be observed directly by microscope, even in the laboratory. Various techniques were come up with to detect these tiny analytes, especially the optical biosensors. Recently, these optical biosensors were applied on smartphone platforms. Taking advantage of the CMOS cameras on smartphones, colorimetric-based and spectrum-based biosensors on smartphone platforms were designed by many groups.

### 3.1. Colorimetric

Smartphone-based colorimetry was acknowledged as an innovating technology, and various applications are under rapid development. The sensing mechanism of colorimetric detection is mainly focused on lateral flow immunoassay (LFIA), enzyme-linked immunosorbent assay (ELISA), chemiluminescence, electrochemiluminescence, Mie scattering and color pattern arrays. In these detection methods, the smartphone camera was crucial to receive accurate detection results. The camera was used to capture images of the reference sample and the target sample, respectively. Then the images were digitized and decomposed into different color spaces that were decided by the specific needs of the assay. The result could be obtained by comparing the target color with the reference. While the color in the image is very sensitive to the light conditions and quality of cameras, several methods were applied to guarantee a reliable result.

Considering that the light conditions always change with the surroundings, it is important for colorimetric-based biosensing to eliminate the influence of background light. Therefore, several methods were applied to eliminate this influence. First, setting a reference sample is an effective method. The reference sample should be measured under same conditions as the target sample to avoid the influence of light conditions. Oncescu et al. proposed a smartphone-based accessory for colorimetric detection [30]. In this work, a disposable test strip was used to detect the pH level in sweat or saliva samples (Figure 3.1). On the test strip, there was a reference strip which was made of white plastic material. This reference strip could help to detect changes in white balance on the camera during analysis. As for the test with a certain reference, the standard reference could be a perfect choice. Shen et al. reported a smartphone-based colorimetric biosensor to detect the pH level [31]. Test strips used in this work was commercially-available pH test paper (Figure 3.2). Therefore, the standard reference paper was provided when purchasing. In this work, both images of reference paper and test strip were taken by smartphone cameras, which usually integrate some automated functions, such as auto white balance. These functions are useful for people to take normal photos, but is necessary for colorimetric analysis. The reference paper could help to eliminate the effects brought by these functions. For some monochrome detections, such as fluorescence-based detection [32] and concentration detection [33], the reference could be simpler. Yetisen et al. presented a fluorescence-based biosensor for tear electrolyte analysis [32]. Different fluorescence was emitted when the different ions in tears reacted with certain materials used in this work (Figure 3.3). The fluorescence intensity at the ion-free concentration and the ion-saturated concentration were detected as the reference. The free ion concentration could be calculated by a formula related to the fluorescence intensity of the test sample. Also, Kim et al. presented a smartphone-based biosensor for colorimetric analysis of hematocrit levels [33]. Since different hematocrit levels could present different brightness, the hematocrit level could be identified through comparing with the blank part of the microfluidic chip. Thus, the reference in this work was just the blank part of the microfluidic chip (Figure 3.4).

Second, using specific light sources could also avoid the change of light conditions. Kim et al. using a white acrylic-imaging box to isolate the microfluidic device from surroundings to avoid the light from outside. The light source was the flash light in the smartphone which would ensure a stable and uniform photographing condition. However, it brought the image burning effect, which means the images are sometimes burnt out during imaging because the flash light is too bright (Figure 3.4). A polydimethylsiloxane (PDMS) light diffuser was used to avoid irregular and intense light. Like Kim’s group, Oncescu et al. also used a smartphone case which was 3D printed using opaque Vera black material to isolate the test strip from variable external light. A PDMS flash diffuser covering the built-in flash of smartphone to develop a stable and uniform light condition (Figure 3.1). Additionally, compared to Kim’s group, Ocescu’s group developed a very exquisite accessory, which was compact and cheap.

As for fluorescence-based biosensors and other faint light detection techniques, such as chemiluminescence-based detection and reflective phantom interface-based detection, additional attachments were applied to help the camera on the smartphone to detect faint light. Cho et al. had used an attachment which was designed to integrate with the specific smartphone [34]. In this work, the authors combined an organ-on-a-chip (OOC) platform with a fluorescence nanoparticle immune-capture/immune-agglutination assay and that made monitoring of the release of γ-glutamyl-transpeptidase (GGT) to the outflow of the OOC and GGT on the apical brush border membrane of 786-0 proximal tubule derived cells (PTCs) possible. An additional LED and battery was used as the excitation source. A microlense and a 500 nm long-pass filter were placed in front of the camera of the smartphone to capture the fluorescence signals (Figure 4.1). To get an accurate result, a fluorescent nanoparticle was used to reduce thr assay time and provide enough specificity. Fluorescent nanoparticles could be used as detection objects and detected by the smartphone-based microscope. In other fluorescence-based detections, a light filter was also used to help detect the fluorescence light with specific wavelengths [32,35]. Roda et al. used a 3D-printed device to fabricate a smartphone-based chemiluminescence biosensor for lactate in oral fluid and sweat [36]. In this work, a disposable analytical cartridge, a mini dark box and a smartphone adapter were used to detect the chemiluminescence derived from enzyme-coupled reaction (Figure 4.2). The dark box avoided the influence of ambient light which is usually brighter than chemiluminescence and could cover the target signal. Also, Lebiga et al. reported a smartphone-based chemiluminescence detection used a paper-plastic disposable microfluidic device (Figure 4.3) [37]. The microfluidic device achieved same function as the dark box used in [36]. Giavazzi et al. using the theory of reflective phantom interface (RPI), which measured the intensity of light reflected by a surface with slight change of reflectivity [38]. A custom-designed cradle was used to help the camera detect the light reflected by a special substrate to detect multiple targets (Figure 4.4). When different antibodies were captured by the substrate, the reflectivity of substrate changed slightly. The reactive groups were immobilized on the solid-liquid interface for capturing the antibodies and preventing undesired reaction. On the light path, apertures and a polarizer were placed, which contributed to reduce background light.

Third, algorithms for data analyzing had also been developed to eliminate the background signal. In [33], a gray-scale-valuation (GSV)-based algorithm was used to distinguish the brightness difference between the reference area and the detection area, while in [30], the red-green-blue (RGB) value and the hue were calculated by the algorithm in the APP. Moreover, Hong and Chang used digitizing colors of a colorimetric sensor array to extend the application for multi-analyte sensing [39]. There was no need for additional devices to help the smartphone improve higher quality images when the authors test the sample for the diagnosis. The built-in camera was used as the detector to evaluate the color changes instead of the naked eyes, which could cause errors due to the light conditions and the personal perception. The application mainly achieves these functions, including automatic identification of position, color measurement of each sensor and conversation of corrected colors to concentration values. The team used the application to automatically recognize paper-based sensors. The algorithm helps the smartphone to improve the color changes without any accessories (Figure 4.5). 

On the other hand, it was not easy to affirm the accuracy of the result. After all, the built-in camera of smartphone was easily affected by the light conditions. Meanwhile, Shen’s group also developed an application for quantifying the colors of colorimetric diagnostic assays [31]. The advantage of this group work over Hong’s was that they made the application applicable under different ambient light conditions for compensation through a calibration algorithm developed for correcting errors caused by the ambient light. The work also demonstrated that the high accuracy of pH measurements was in the range of 1–12. Besides, instead of directly using RGB color space, they used the International Commission on Illumination (CIE) 1931 color space for quantification of multiple elements in colorimetric diagnostics. Chromaticity values was used to construct calibration curves of analyte concentrations. Compared work between Hong’s and Chen’s, it illustrated that the technique was applicable to various common light sources, such as sunlight, fluorescent light, or smartphone LED lights. Ultimately, the entire approach could be integrated in a special ‘APP’ to enable one-click reading, making our smartphone based approach operable without any professional training or complex instrumentation. Meanwhile, the advantages of inexpensive and convenient paper-based colorimetry and the ubiquitous smartphone were tied to achieve a ready-to-go POC diagnosis.

These systems had several characteristics including a reference color chart, paper-based biosensors, the smartphone built-in camera as the detector, and an algorithm for data analysis and signal improvement. The most important function of these systems was digitizing the image to obtain quantitative results to avoid the personal errors of naked eyes. However, colorimetric measurements had its own drawbacks, these systems rely on a reference color chart, which was the basis of the comparison process. Even so, we still believed the colorimetric detection technology could be broadly applied to POC diagnosis with any type of colorimetric test strip, or to any sensor systems that provided colorimetric response.

The detailed information and the comparison of all the biosensor based on colorimetric on smartphone platform is shown in the Table 2.

### 3.2. Spectrum

Using smartphones to detect the color change is more precise than naked eyes due to the digital processing of images. However, the CMOS camera of smartphones could record more information than color which classified by the RGB or HVS model. The detail information of light record by smartphones could be presented as spectrum. The smartphone-based spectrometry could be used in various methods of biosensing, such as surface plasmon resonance (SPR), Localized surface plasmon resonance (LSPR) and other absorbance or reflectance based biosensing.

SPR is a relatively mature technology. There are many advantages of this label-free technique over other methods. SPR is suitable for turbid, non-transparent or colored solutions, and it is capable of real-time continuous monitoring of the reaction process. Besides, SPR is a convenient and quick detection method. These characteristics provide wide application prospects. A SPR-based platform has been reported by Lepage et al. for real-time detection of influenza virus [40]. Then et al. presented an assay for quantitative detection of weak d antigen variants in blood by using the SPR technology [41]. SPR technology is also used in measuring the relative permittivity of a physiological solution [42], measuring antibodies binding to a functionalized sensing element [43], and quantitative determination of multiple tumor markers [44].

SPR has been widely explored for its high sensitivity to refractive index changes caused by the interactions of detected molecules with the molecules immobilized on the surface of the biosensors. Though many SPR biosensing methods provide label-free and real-time detection, it just offers single-spot SPR sensing and this has limited the SPR use for high-throughput and multiplexed detection. To achieve multi-analyte detection, Guner et al. developed a smartphone-based surface plasmon resonance imaging (SPRi) platform [45]. Unlike the traditional sensors, multiple sensing points were designed and fabricated on the biosensor chip. The authors also used refractive index changes to measure the analytes (Figure 5.1). This system has its own advantages, including the capacity of detecting multiple analytes, and containing multiple sensing points which enabled serial dilution assays. Besides, the group achieved replicated measurements on the same sensor chip and converted the Blu-ray discs to plasmonic surfaces, and both designs lower the cost of the assay. To demonstrate the performance of the SPRi platform, this group used the device to detect mouse IgG antibodies. In the process, many images were recorded for analysis and data processing. The data were converted to the intensities which could be related to the refractive index.

Wang et al. demonstrated a self-referenced portable SPR imaging platform for biosensing [46]. An unique nanostructure named nanoLCA (Nano Lycurgus cup array) was formed to serve as a sensing platform based on two sensing principles: liquid refractive index sensing and optical absorbance enhancement sensing. Based on the imaging and color analysis system on smartphones, the spectrum of transmission were recorded. The change of the spectrum indicated the change of liquid refractive index. Standard square color patterns and a black plastic sheet were used to eliminate the interference of ambient light. (Figure 5.2).

Moreover, based on the theory of plasmonic resonance, researchers changed the light source to enhance the limit of detection (LOD). When the light source has a similar wavelength as the absorbance peak wavelength of the nanoLCA, the LOD of this sensor could be 100 times more sensitive than that under a white light source. This work discussed the effect of light source, and came up with a method that using absorbance enhancement to promote the LOD of the sensor based on SPR.

LSPR is based on the measurement of the peak shift of the extinction spectrum or reflection spectrum, which was different from the theory of SPR detection. Though SPR possess better refractive index sensitivity than LSPR, the requirement for temperature control equipment added to the cost and complexity of the SPR system.

On the contrary, the LSPR biosensors were not sensitive to the ambient temperature. Therefore, the experimental device was simpler than the SPR. In addition, the LSPR did not require this film deposition which lowed the cost. By taking advantage of the LSPR biological sensing technology, ultra-small-volume, simple-system-setting, convenient-operation and label-free systems could be obtained.

The first smartphone-based LSPR spectrophotometer for bio-conjugation detection and quantification was developed by Dutta [8]. The smartphone-based LSPR sensor platform could be used for recording and analyzing LSPR spectra which were determined by the size change of metal nanoparticles and caused by the biomolecular conjugation with metal nanoparticles. The spectrophotometer consists of a smartphone, some optic components, custom-designed cradle and an external broadband light source (Figure 5.3). To demonstrate the performance of the smartphone-LSPR platform, the spectrophotometer was used to quantify the binding of bovine serum albumin (BSA) with gold nanoparticles and the attachment of trypsin enzyme to Au nanoparticles (AuNP) surface. The variation of the size of the AuNPs and magnitude of biomolecules attached to it could be quantified by measure the shift in LSPR peak absorption wavelength. The detected results were compared with the laboratory spectrophotometer which proved the high reliability of the platform. Though this platform could be applied for an alternative hand-size LSPR sensing platform, it had its own drawbacks. For example, the dispersed spectrum of the broadband light source was recorded by the smartphone and then transferred to the desktop that executes the post-image processing. This would limit the platform to be a portable easy-to-use platform. To overcome the drawbacks, another LSPR-based smartphone spectrometer which could be used for colorimetric biosensing assays was also developed [47]. In this work, the authors simplified the optics used in the smartphone-based spectrometer including lens and filters (Figure 5.4). To lower the cost, a compact disk (CD) with a grating was used as the dispersive unit. Besides, the smartphone built-in LED was used as the light source which fixed the instability and requirement for complex electric circuit of the external light source. To demonstrate the performance of the smartphone spectrometer, the authors used the device to detect the glucose and human cardiac troponin I (cTnI), respectively, and the LOD are both better than the plate-read or UV/Vis spectrometer. Compared to commercial plate-readers, the developed smartphone spectrometer shows a higher sensitivity. What’s more, the device achieved real time monitoring of the peak position of the LSPR absorbance spectra whereas the commercial plate-reader does not.

Apart from SPR and LSPR, other absorption and reflection-based biosensing technologies were also applied on smartphones. An example was presented by Gallegos et al. who developed a smartphone-based miniaturized spectrometer for label-free detection [48]. The authors used optical components to adjust the light, a custom designed cradle to hold the optical components and a smartphone (Figure 5.5). A diffraction grating of the optical components is used to separate the visible light corresponding to different wavelengths. The system was used to measure the transmission spectrum of the photonic crystal (PC) biosensor. To detect the analytes, the authors measured the shifts in the resonant wavelength of PC. When the analytes in the test sample was adsorbed by the capture molecule immobilized on the PC, the resonant wavelength changed due to the change of the refractive index of the PC. The authors demonstrate the performance of the label-free detection system by measuring the spectrum of PC immobilized with protein A before and after the addition of porcine immunoglobulin G (IgG). This system could also be used to detect other antibodies or molecules interested. However, this system also has its drawbacks. In the experiment process, surface of the PC biosensor was required to keep a dry state.

### 3.3. Summary

Spectrometry-based biosensord on smartphone platforms were mainly using the function of the CMOS array that is integrated in smartphones. When the optical signal was recorded by the CMOS array, the information of the signal could be presented after processing by software. Among these spectrometry-based biosensors, colorimetric-based biosensors represent cheap techniques that extract the characteristics of several colors based on the theory of color space. Colorimetric-based biosensors were usually used to detect reactions which produced significant changes in color. However, these significant changes in color meant that plenty of analyten was required. In many cases, the concentration of analytes is very low, the change in color is not significant enough to be detected. Therefore, spectrum-based biosensors were designed to detect analytes with an extremely low concentration. The spectrum-based biosensor could record the intensity of different wavelengths of light, and the changes of spectrum mean some reaction has happened or some analytes are present. The spectrum-based biosensor on smartphone platforms used the CMOS array to monitor the changes of spectrum of light signal. These spectrum-based biosensors were very sensitive to analytes. However, they were sensitive to the surroundings too. The temperature, humidity and even nanoparticles existing in surroundings could influence the spectrum. Therefore, a considered algorithm was needed to eliminate these influences. Besides, the CMOS array integrated in smartphones were not as precise as spectrometers used in the laboratory. High sensitivity chips for these spectrum-based biosensors were also needed to enlarge the changes of spectrum which could be identified by smartphones. Unlike the colorimetric-based biosensor, high-sensitive chips for spectrum-based biosensors were expensive and needed complex fabrication processes. Cost and function are always a trade-off business. To break this trade-off, the techniques of fabrication and the theory of detection should be developed further. All biosensor based on spectrum on smartphone platform are compared in the Table 3.

## 4. Conclusions

This review has presented a summary of smartphone-biosensor platforms based on optical theory such as imaging, absorbance, reflectance, fluorescence, surface plasmon resonance (SPR), localized surface plasmon resonance (LSPR), which could be roughly classified into: (1) Image biosensors (chemiluminescence (CL), gray-scale-valuation (GSV), red-green-blue (RGB), hue-saturation-value (HSV)), which used integrated cameras for taking the picture of results and through the APP to analyze it or get other optical information; (2) Spectrum biosensors (surface plasmon resonance (SPR), localized surface plasmon resonance (LSPR), photonic crystal (PC)) integrated with smartphone or microfluidic chip, which are optical detection methods for quantifying bio-molecular interactions with the help of the optical properties of nanostructures. Those biosensors based on smartphone could provide excellent mobile platforms to control, perform, analyze, and display sensor processes for applications ranging from fundamental researches to practical point-of-care detection tasks, such as cell and DNA imaging, serum diagnosis, and environmental monitoring.

Meanwhile, among these smartphone-based biosensors, optical biosensing techniques were widely applied due to the high quality of the built-in camera of smartphones. The built-in camera served as the detector what was usually a spectrometer or a microscope in the laboratory. The photos or videos taken by the built-in camera were processed by APPs on smartphones or software on a computer. Based on the special theory and mathematic models, the data were extracted from the photos. After analyzing the data, sensing results were brought out. With the help of smartphone-based biosensors, which are available for many people, and some cheap attachments, people could complete some simple tests in home or in the wild. These simple tests could help people monitor their physical condition. Also, these tests could help doctors make decisions about treatments when professional equipment is lacking.

However, to commercialize the smartphone-based analytical devices, there are still many challenges in the smartphone-based platforms. Firstly, optical-based biosensors are sensitive to the change of ambient conditions. However, attachments are applied for almost all smartphone-based optical biosensors. The design of a convenient and reliable attachment should be considered. Secondly, the attachment-free smartphone-based optical biosensor could also be taking into account. Second, the software on smartphones should be easy-to-use. Researchers should realize that the software is operated by the non-professionals, the operation should be as simply as possible and the results should be as clear as possible. Thirdly, the hardware of smartphones from different manufacturers varies, which may lead to different sensing results. More tests should be taken to justify that a smartphone-based biosensor is suitable for most smartphones.

Therefore, with the development of intelligent handset technology and the extensive research of the global specialists, we believe the smartphone-based POC testing devices will become more and more convenient and usable in the near future. Especially, for the optical biosensors based on smartphone platforms, the biosensing signals are often transmitted by optical attachments, components and digital cameras in the form of images. Then, the image information could be analyzed by smartphone or delivered to the servers through wireless networks. Lastly, much effort should be taken to minimize optical attachments for smartphones by simplification of optical paths and components for small size and light weight.

## Figures and Tables

**Figure 1:**
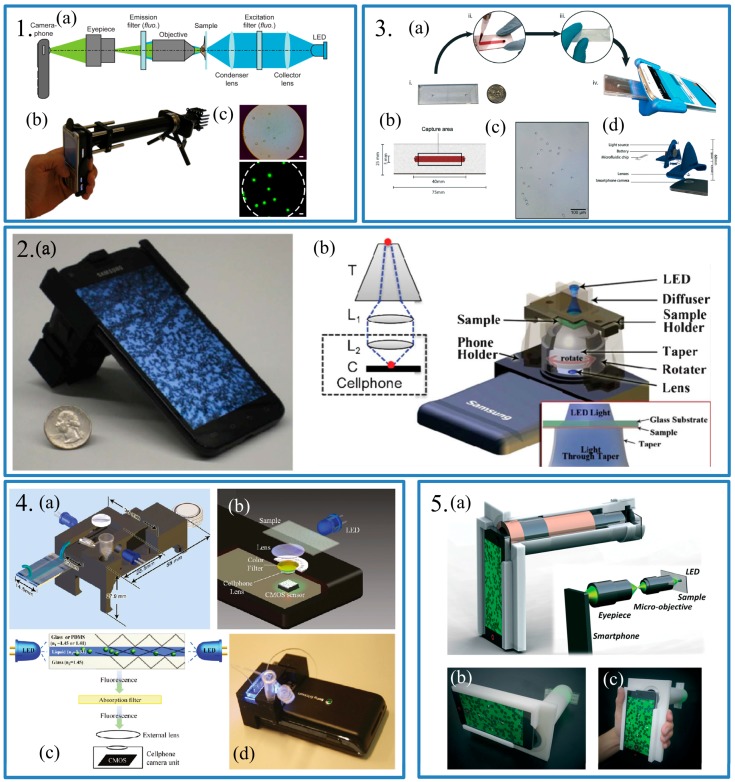
**Panel 1:** (**a**) Layout of the smartphone-based microscope for fluorescence imaging. The same apparatus was used for bright field imaging, with the filters and LED removed. Components only required for fluorescence imaging were indicated by “fluo”. (**b**) A prototype, with filters and LED installed, capable of fluorescence imaging; (**c**) Bright field image and (**d**) fluorescent image of 6 μm fluorescent beans. Scales bars are 10 μm. Reproduced from [20]. **Panel 2:** (**a**) Photograph of the Contact Scope installed on an Android smartphone; (**b**) Schematic diagram of the smartphone attachment of the Contact Scope. Reproduced from [21]. **Panel 3:** (**a**) The process flow showing the steps involved in performing the assay; (**b**) The dimensions of the disposable microfluidic chip; (**c**) The image of captured CD4 cells on-chip using the smartphone system; (**d**) The exploded image of the smartphone attachment and the relevant dimensions. Reproduced from [22]. **Panel 4:** (**a**) The dimensions; (**b**) The schematic diagram and (**c**) The working mechanism of the designed optical attachment for optofluidic fluorescence imaging cytometer on a smartphone; (**d**) The photograph of the optofluidic fluorescent imaging cytometer on a smartphone. Reproduced from [23]. **Panel 5:** (**a**) Design and (**b**) optical system of the smartphone based hand-held quantitative phase microscope; (**c**) Integrated smartphone based hand held quantitative phase microscope with its 3-D printed shell. Reproduced from [1].

**Figure 2:**
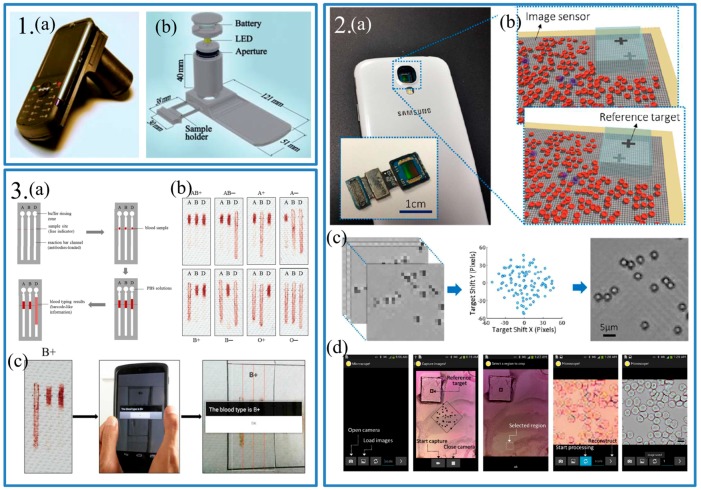
**Panel 1:** (**a**) The photograph of the lens-free smartphone microscope; (**b**) Schematic diagram of the microscope attachment shown in (**a**). Reproduced from [27]. **Panel 2:** (**a**) The photograph of the prototype device which was an Android smartphone with the lens module of the camera removed. The inset showed the image sensor module with the lens removed; (**b**) The working mechanism of the smartphone-based microscope. The shadow of a reference target and samples in different angle were traced when the user tilts the device around; (**c**) The reconstructed process took placed in the smartphone. 100 raw images (**Left**) were taken to get a reconstructed image (**Right**); (**d**) Imaging process of with the custom-built application. From left to right: starting to capture images or load images, capturing images, selecting a region to reconstruct, starting processing of reconstruction, viewing result and saving. Reproduced from [28]. **Panel 3:** (**a**) A schematic diagram of the barcode-like blood typing device. Along the arrow: introducing the antibodies into the reaction bar channels, introducing the blood sample into the reaction region, eluting the channels with PBS solutions, reading the blood typing test results; (**b**) The actual assays of all eight ABO/RhD blood types by the optimized barcode-like paper based blood typing device; (**c**) Process of the smartphone-based blood typing device. Along the arrow: getting the blood typing test result (B+), reading the result using an Android application, obtaining the blood result with text on the screen. Reproduced from [29].

**Figure 3:**
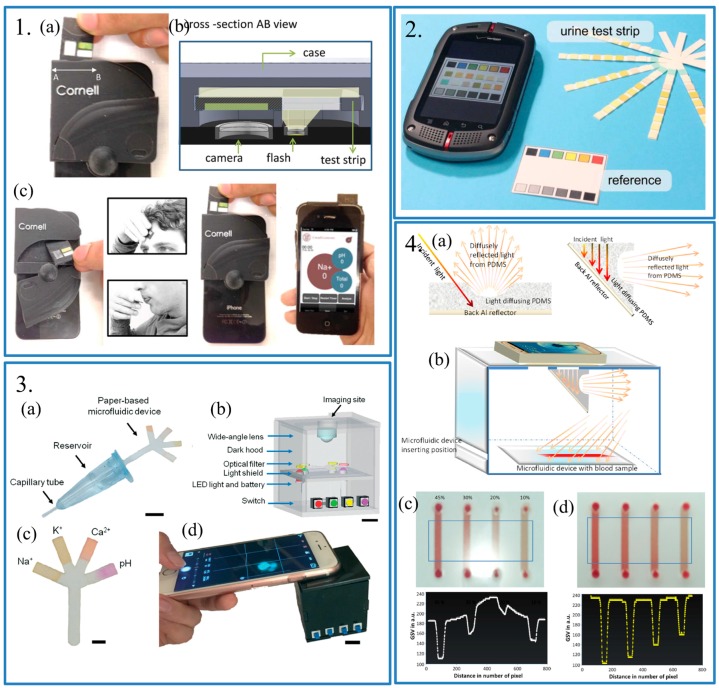
**Panel 1:** (**a**) Photograph of the test strip and its insertion; (**b**) The cross-section view of the optical system; AB in both (**a**) and (**b**) was the same place; (**c**) Working processing of the smartphone-based health accessory for colorimetric detection of biomarkers in sweat and saliva. From left to right: removing a test strip form the back-storage compartment, acquiring sweat or saliva samples, inserting the test strip in the optical system, analyzing the pH using the APP. Reproduced from [30]. **Panel 2:** Photograph of the devices for colorimetric detection. A smartphone, a urine test strip and a reference. Reproduced from [31]. **Panel 3:** (**a**) Schematic of the sample collection and dilution device. Scale bar = 1 cm; (**b**) Schematic of the portable readout device. Scale bar = 1 cm; (**c**) Photograph of the paper-based microfluidic device impregnated with fluorescent probes. Scale bar = 2 mm; (**d**) Photograph of the using of the paper-based microfluidic system for tear electrolyte analysis. Scale bar = 4 mm. Reproduced from [32]. **Panel 4:** (**a**) Illustrate of the light diffusing effect using a U-shaped PDMS placed on a reflecting aluminum back contact; (**b**) Schematic side view of the smartphone-based optical platform for colorimetric analysis. Images of different hematocrit samples and their grayscale value (**c**) without and (**d**) with PDMS light diffuser. Reproduced from [33].

**Figure 4:**
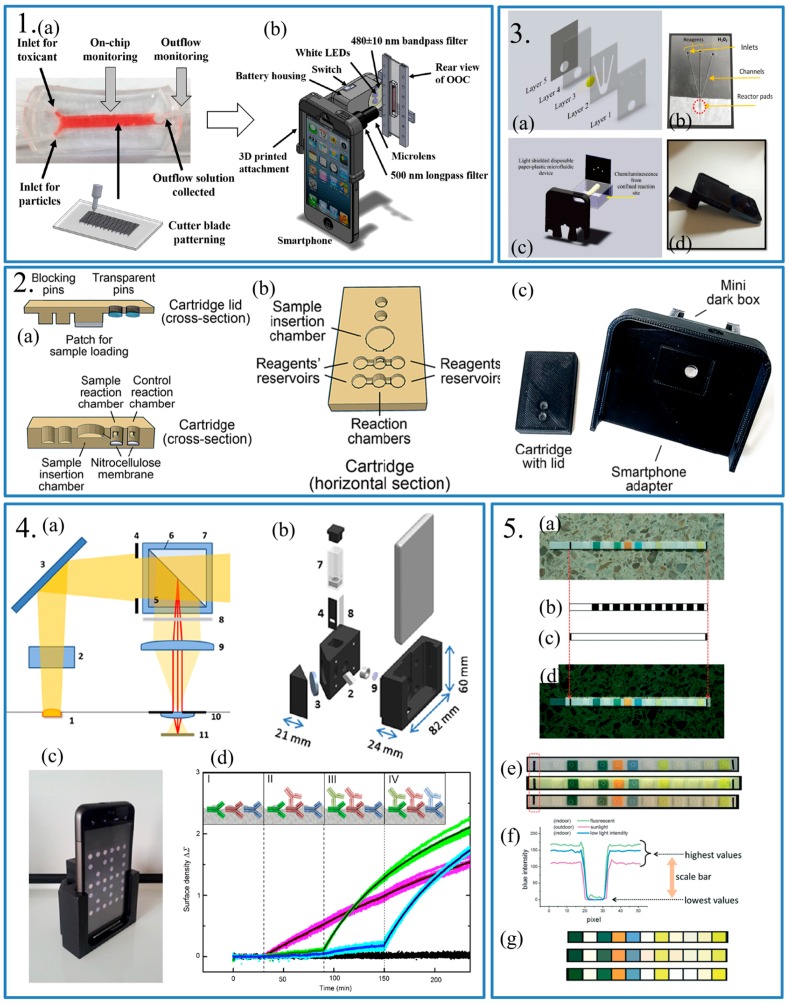
**Panel 1:** (**a**) Photograph of the microfluidic device and schematic of cutter blade patterning; (**b**) Schematic of the dual-mode monitoring of organ-on-a-chip with smartphone based fluorescence microscope. Reproduced from [34]. **Panel 2:** (**a**) The cross-section schematic of the cartridge lid and cartridge; (**b**) The horizontal section schematic of the cartridge; (**c**) Photograph of the cartridge-lid assembly and of the min dark box and smartphone adapter. Reproduced from [36]. **Panel 3:** (**a**) Exploded view and (**b**) Photograph of the custom-designed paper-plastic disposable microfluidic device; (**c**) Schematic of the smartphone accessory for video capture of chemiluminescence reaction. (**d**) Photograph of the smartphone-based confined chemiluminescence detection device. Reproduced from [37]. **Panel 4:** (**a**) Schematic and (**b**) Exploded view of the optical elements contained in the plastic cradle; (**c**) Photograph of the smartphone-based label-free immunoassay; (**d**) The surface density measured by the smartphone. The times of the target additions were indicated as vertical dashed lines. Inset: the binding of the target antibodies in solution to the immobilized IgG is schematically represented before and after the additions (I-IV). Reproduced from [38]. **Panel 5:** (**a**) An optical image of a urine strip consisting of 12 paper-based sensors in array; (**b**,**c**) The mono-color template using to estimate the position of each sensor array; (**d**) Image resulting from the automatic recognition; (**e**) Images of a urine strip under different light condition, from up to down: indoor fluorescent light, outdoor sunlight and indoor low light intensity conditions; (**f**) The blue color profiles of the black and white background were measured in the dotted red rectangle in (**a**,**g**). The corrected colors were obtained from the colors of (**a**) by the correction protocol. Reproduced from [39].

**Figure 5:**
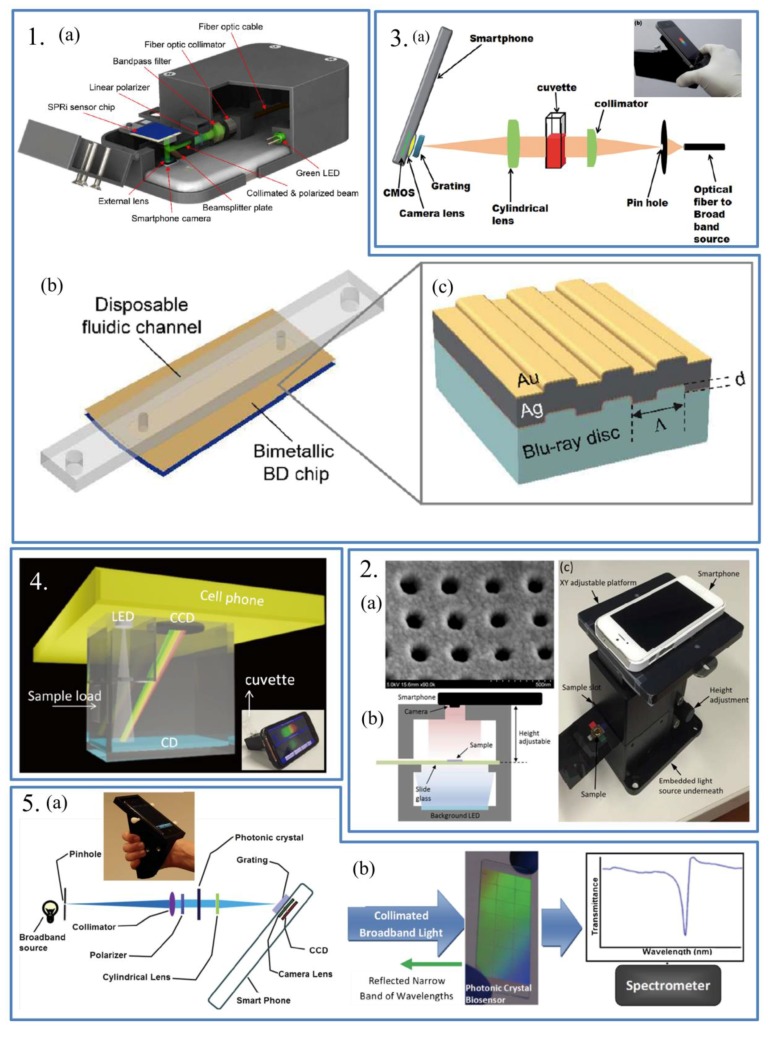
**Panel 1:** (**a**) Schematic of the smartphone based SPRi platform; (**b**) Schematic of the SPRi chip which integrated a bimetallic Blu-ray disc and a disposable fluidic channel; (**c**) Schematic of the bimetallic Blu-ray disc. Reproduced from [45]. **Panel 2:** (**a**) SEM image of nanoLCA; (**b**) Schematic of internal optical detection system setup; (**c**) Real image of optical detection setup. Reproduced from [46]. **Panel 3:** (**a**) Schematic of the smartphone-based LSPR sensing platform. (**b**) Insert: photograph of the smartphone-based LSPR sensing platform. Reproduced from [8]. **Panel 4:** Schematic of the smartphone spectrometer for colorimetric biosensing. Insert: Photograph of the smartphone spectrometer. Reproduced from [47]. **Panel 5:** (**a**) Schematic of the optical components within the smartphone cradle; (**b**) Working principle of the smartphone-based photonic crystal biosensor. When the photonic crystal was illuminated with a wide range of wavelength, only a narrow band of wavelengths was reflected, while the spectrometer recorded all other wavelength transmitted through the photonic crystal. Insert: Photograph of the cradle with a photonic crystal biosensor slide inserted into the detection slot. Reproduced from [48].

**Table 1:** Biosensor based on imaging on smartphone platform.

Sensing Mechanism	Detect Target	Limit of Detection	Smartphone Model	Accessory	Reference
**Brightfield and fluorescence**	Red blood cells (brighfield) sputum (fluorescence)	≥1.2 μm	Nokia N73 3.2 megapixel	LED, filter(for flourescence), lenses, eyepiece, objective	[20]
**Contact microscope**	White blood cells	≥1.6 μm (Samsung) ≥1.5 μm (Sony-Ericsson)	Samsung Galaxy S2, 8 megapixel; Sony-Ericsson Aino, 8 megapixel	LED, lenses, rotater, fiber-optic taper	[21]
**Brightfield**	CD4+ T-cells	≥60 cells per μL	MotoX-XT1575, Motorola	Functionalized microfluidic chip, light source, lenses	[22]
**Fluorescent**	White blood cells	~2 μm	Sony-Erickson U10i Aino, 8 megapixel	Microfluidic chip, LED, plastic color filter, batteries, lens	[24]
**Quantitative phase microscope**	Red blood cells, pap smear, monocot root, broad bean epidermis	-	Nubia Z9 mini	3D printed shell, eyepiece, micro-objective, LED, precision translation stage	[1]
**Lensfree microscopy**	Red blood cells, white blood cells, platelets, Giardia lamblia cysts	≥2.2 μm	Moto Zine ZN5, 5 megapixel	LED, plastic components, battery	[27]
**Lensfree Shadow imaging**	Blood cells, microorganisms	≥500 nm	Samsung galaxy S4, 13megapixels	-	[28]
**Lensfree Image recognition**	Paper-based blood typing device	-	Google Nexus 5	-	[29]

**Table 2:** Biosensor based on colorimetric on smartphone platform.

Sensing Mechanism	Detect Target	Limit of Detection	Smartphone	Accessory	Reference
**Colorimetric**	Sweat pH	-	iPhone 4 and 4S	3D printed shells, flash diffuser, test strip	[30]
**Colorimetric**	pH	~0.5 unit (pH)	HTC and BlackBerry	Test strip, reference strip	[31]
**Colorimetric**	Electrolytes in tear	1.0 mmol/L (Na^+^); 1.3 mmol/L (K^+^); 0.02 mmol/L (Ca^2+^); 0.13 unit (pH)	iPhone 6S	Paper-based microfluidic	[32]
**Colorimetric**	Blood (concentration of hematocrit)	0.1%	Galaxy S II	PDMS light diffuser, microfluidic device, PMMA box	[33]
**Fluorescence**	proteins	10 pg/mL	iPhone 5S	LED, 3D printed attachment, organ-on-a-chip	[34]
**Chemiluminescence**	Lactate levels in oral fluid and sweat	0.5 mmol/L (oral fluid); 0.1 mmol/L (sweat)	Samsung Galaxy SII Plus	3D printed analytical device	[36]
**Chemiluminescence**	H_2_O_2_	250 nmol/L	iPhone	Disposable paper-plastic microfluidic device	[37]
**Reflective Phantom Interface**	Hepatitis B and HIV	10 ng/mL	HTC DesireHD	Plastic cradle	[38]

**Table 3:** Biosensor based on spectrum on smartphone platform.

Sensing Mechanism	Detect Target	Limit of Detection	Smartphone	Accessory	Reference
**SPR imaging**	Mouse IgG	A few nmol	SamsungI8552 Galaxy Win	Disposable fluidic chip, Bimetallic BD chip, Optical attachment	[45]
**SPR and LSPR**	BSA	0.01 mg/mL	iPhone 6	Adjustable platform, LED, nano Lycurgus cup array chip	[46]
**LSPR**	BSA	19.2 μg/mL	iPhone 4	Lenses, broad band source, grating	[46]
**LSPR**	Cardiac troponin I (cTnI)	50 ng/mL	HTC sensation XE iPhone 5s, Nokia Lumia 920	CD grating, peptide-functionalized AuNPs, shells	[47]
**Photonic crystal**	IgG	4.25 nmol/L	iPhone 4	optical components, broadband source, photonic crystal, grating, pinhole	[48]
